# Outer membrane vesicles containing OmpA induce mitochondrial fragmentation to promote pathogenesis of *Acinetobacter baumannii*

**DOI:** 10.1038/s41598-020-79966-9

**Published:** 2021-01-12

**Authors:** Varnesh Tiku, Eric M. Kofoed, Donghong Yan, Jing Kang, Min Xu, Mike Reichelt, Ivan Dikic, Man-Wah Tan

**Affiliations:** 1 Department of Infectious Diseases, Genentech Inc., 1 DNA Way, South San Francisco, CA 94080 USA; 2 Department of Translational Immunology, Genentech Inc., 1 DNA Way, South San Francisco, CA 94080 USA; 3 Department of Pathology, Genentech Inc., 1 DNA Way, South San Francisco, CA 94080 USA; 4 Institute of Biochemistry II, Faculty of Medicine, Goethe University Frankfurt, Theodor-Stern- Kai 7, 60590 Frankfurt am Main, Germany; 5 Buchmann Institute for Molecular Life Sciences, Goethe University Frankfurt, Max-von-Laue-Str. 15, 60438 Frankfurt am Main, Germany; 6 Max Planck Institute of Biophysics, Max-von-Laue-Str. 3, 60438 Frankfurt am Main, Germany

**Keywords:** Microbiology, Pathogens

## Abstract

*Acinetobacter baumannii* is a highly antibiotic resistant Gram-negative bacterium that causes life-threatening infections in humans with a very high mortality rate. *A. baumannii* is an extracellular pathogen with poorly understood virulence mechanisms. Here we report that *A. baumannii* employs the release of outer membrane vesicles (OMVs) containing the outer membrane protein A (OmpA_Ab_) to promote bacterial pathogenesis and dissemination. OMVs containing OmpA_Ab_ are taken up by mammalian cells where they activate the host GTPase dynamin-related protein 1 (DRP1). OmpA_Ab_ mediated activation of DRP1 enhances its accumulation on mitochondria that causes mitochondrial fragmentation, elevation in reactive oxygen species (ROS) production and cell death. Loss of DRP1 rescues these phenotypes. Our data show that OmpA_Ab_ is sufficient to induce mitochondrial fragmentation and cytotoxicity since its expression in *E. coli* transfers its pathogenic properties to *E. coli*. *A. baumannii* infection in mice also induces mitochondrial damage in alveolar macrophages in an OmpA_Ab_ dependent manner. We finally show that OmpA_Ab_ is also required for systemic dissemination in the mouse lung infection model. In this study we uncover the mechanism of OmpA_Ab_ as a virulence factor in *A. baumannii* infections and further establish the host cell factor required for its pathogenic effects.

## Introduction

*Acinetobacter baumannii* is an aerobic, pleomorphic Gram-negative bacterial pathogen that causes severe infections including pneumonia and bloodstream infections that are often fatal^1,2^. Coupled with high prevalence of multidrug resistant phenotypes, it is now considered as “priority one pathogen” by the World Health Organization (WHO) for which new antibiotics and alternative treatment options are rapidly needed^3^. Strategies employed by *A. baumannii* to resist antibiotics include the expression of β-lactamases^4^, multidrug efflux pumps^5^, and aminoglycoside-modifying enzymes^6^. Another major attribute leading to widespread persistence of *A. baumannii* in nosocomial environments is its ability to withstand harsh conditions that can prove to be inhospitable for other pathogens thus providing *A. baumannii* with a survival advantage. It can withstand disinfection^7,8^, long periods of desiccation^9,10^ and oxidative stress^11^. Additionally, *A. baumannii* forms robust biofilms within the host that can further contribute to infection. Survival within these biofilms enhances extracellular stress tolerance and thereby augments bacterial persistence^12,13^. Even though some of these microbial properties of *A. baumannii* have been fairly well studied, its interaction with the host and the mechanisms that it employs to damage host-defense remain poorly understood.

One of the important virulence factors of *A. baumannii* is the outer membrane porin called outer membrane protein A (OmpA_Ab_). High expression of OmpA_Ab_ is a potential risk factor for enhanced mortality in humans infected with *A. baumannii*^14^. OmpA is a highly abundant ß-barrel porin protein embedded in the outer membrane of Gram-negative bacteria. The protein consists of extra-cellular loops protruding outwards and is non-covalently anchored to peptidoglycan in the bacterial periplasm^15^. OmpA forms a non-selective channel in bacterial outer membranes that allows for the passage of ions and other solutes^15,16^. OmpA_Ab_ serves multiple functions in *A. baumannii,* including resistance against complement killing and biofilm formation^17,18^. *A. baumannii* virulence factors including OmpA_Ab_ and certain tissue degrading enzymes are delivered to host cells via outer membrane vesicles (OMVs)^19^. Purified recombinant OmpA_Ab_ protein has been reported to colocalize with mitochondria^20^. However, it is not known if OMVs carrying OmpA_Ab_ target host mitochondria in an actual infection with *A. baumannii*. Moreover, the host factors that promote the effects of OmpA_Ab_ intracellularly remain elusive. Also, the impact of OmpA_Ab_ on mitochondrial morphology and function during infection are unknown.

Mitochondria are pivotal in maintaining cellular homeostasis by regulating a host of major cellular activities ranging from metabolism and energy production to immune function and cell death. Mitochondria have a very dynamic morphology switching back and forth between fused tubular networks to fragmented rounded bodies depending on the stimulus^21,22^. These morphological states are associated with specific functions. Morphology of mitochondria is regulated by dynamin-related GTPase proteins—Mitofusin 1 and 2 (MFN1 and 2) that induce mitochondrial fusion and DRP1 (also called DNM1L), which promotes mitochondrial fission leading to fragmentation of the mitochondrial network. Given the central role that the mitochondria play in regulating diverse cellular functions, many pathogens have evolved ways of targeting mitochondria to perturb cellular defense mechanisms and enable their intracellular replication^23–25^. A handful of bacteria, including *H. pylori* and *L. pneumophila*, have also been shown to induce mitochondrial fragmentation in a DRP1 dependent manner^24^.

Here we describe the mechanism by which *A. baumannii* utilizes OmpA_Ab_ to inflict host cell damage during infection. Using genetic and cell biological approaches we demonstrate that outer membrane vesicles (OMVs) containing OmpA_Ab_ localize to host cell mitochondria and induce mitochondrial fragmentation and cytotoxicity. *E. coli* heterologously expressing OmpA_Ab_ also induces host cell mitochondrial fragmentation suggesting that OmpA_Ab_ is sufficient to induce this phenotype. Mechanistically OmpA_Ab_ activates the host GTPase protein DRP1 which colocalizes with mitochondria and induces mitochondrial fragmentation and cytotoxicity. Loss of DRP1 rescues these phenotypes. Using a mouse intra-nasal lung infection model, we show that OmpA_Ab_ induces mitochondrial fragmentation in alveolar macrophages and systemic bacterial dissemination from the lungs to other organs. Together, these observations provide mechanistic insights by which OmpA_Ab_ contributes to *A. baumannii* pathogenesis in vitro and in vivo.

## Results

### OmpA_Ab_ is required for bacterial colonization and dissemination in vivo

OmpA_Ab_ has been reported to be an important virulence factor in *A. baumannii*, however its role in in vivo infections is not completely understood. To investigate the role of OmpA_Ab,_ we generated OmpA_Ab_ mutants in *A. baumannii* by replacing the endogenous OmpA_Ab_ with a kanamycin cassette. 500 base pairs of flanking sequences up- and downstream of OmpA_Ab_ served as homology arms for genetic recombineering to generate the ΔOmpA mutant strain (Supplementary Fig. 1A,B)^26^. The ΔOmpA mutant strain was verified by Sanger sequencing. A complemented strain was generated by adding the flag-tagged wildtype copy of OmpA_Ab_ into the ΔOmpA mutant strain. OmpA_Ab_::Flag expression was validated by Coomassie staining and western blotting (Supplementary Fig. 1B,C). Addition of the flag tag did not interfere with the function of OmpA_Ab_ since the flag-tagged complemented strain completely recapitulated OmpA_Ab_ associated phenotypes in the ΔOmpA mutant strain (shown below).

**Figure 1:**
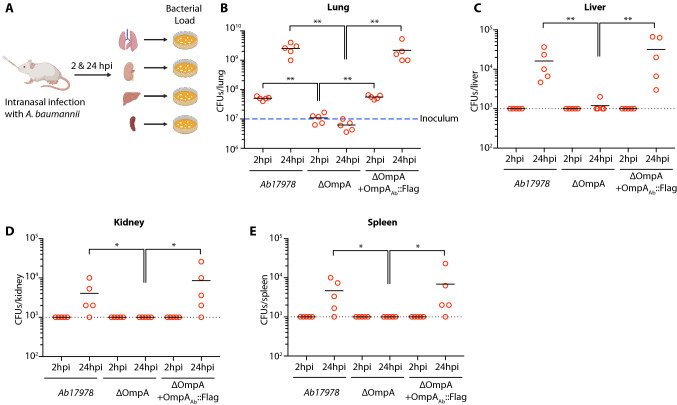
OmpA_Ab_ is required for bacterial colonization and dissemination in vivo*.* (**A**) Schematic representation of the mouse infection (created with BioRender.com). (**B**–**E**) Neutropenic BALB/c mice were infected intranasally with 10^7^ colony forming units (CFUs) of the indicated bacteria. Each group included five mice per time point. Two- and 24-h post-infection, animals were euthanized and lungs, liver, kidneys and spleen were homogenized and CFUs were plated from these organs. Blue dashed line in (**B**) represents the CFU with which each mouse was infected with. Black dotted line in (**C**–**E**) represents the limit of detection. Shown is the representative data from two independent experiments. Two-tailed *p* value using Mann–Whitney test *p $\le$ 0.05, **p $\le$ 0.01.

Since the lung is the primary site of *A. baumannii* infection^2,27^, we investigated the role of OmpA_Ab_ using the intranasal mouse lung infection model (Fig. 1A). We infected BALB/c mice intranasally with 10^7^ CFUs of wildtype *A. baumannii Ab17978*, isogenic *A. baumannii* ΔOmpA and the ΔOmpA + OmpA_Ab_::Flag complemented strain. At 2- and 24-h post-infection, bacterial load was assessed to evaluate bacterial colonization in lungs. Wildtype *A. baumannii Ab17978* and the ΔOmpA + OmpA_Ab_::Flag complemented strain grew by over 2-logs in the lungs in the course of 24 h. By contrast no outgrowth beyond the initial inoculum was observed for *A. baumannii* ΔOmpA in the lungs (Fig. 1B). We also determined the ability of these strains to disseminate to other organs at 24-h post-infection. Wildtype *A. baumannii Ab17978* and the ΔOmpA + OmpA_Ab_::Flag complemented strain could be detected in the liver, kidneys and spleen (Fig. 1C–E), indicating the ability of these strains to disseminate and cause a systemic infection. Despite the ability of *A. baumannii* ΔOmpA to partially persist in the lung, *A. baumannii* ΔOmpA was below the limit of detection in all the organs tested (Fig. 1C–E). These results demonstrate that OmpA_Ab_ is required for bacterial dissemination from the mouse lung during an infection. These results are also suggestive of the role of OmpA_Ab_ in the disruption of the lung epithelial barrier which is required for bacterial dissemination to other organs.

### OmpA_Ab_ is necessary and sufficient to cause mitochondrial fragmentation

**Figure 2:**
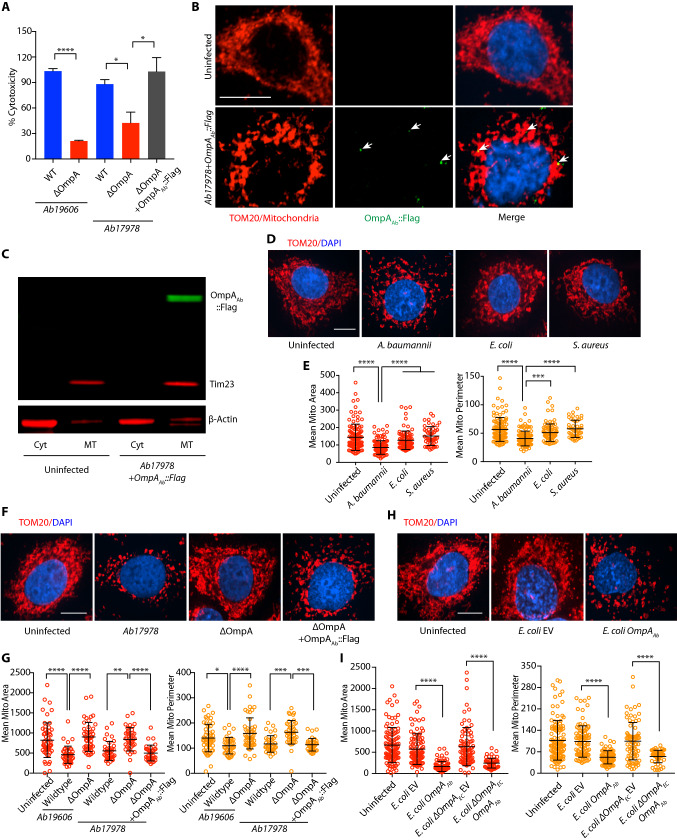
OmpA_Ab_ is necessary and sufficient to cause mitochondrial fragmentation. (**A**) Cytotoxicity was assessed by LDH release assay in A549 cells treated with the indicated bacteria at MOI 50 for 24 h. The experiments were done in triplicates. Error bars represent standard deviation. Two-tailed *p* value using unpaired t-test *p $\le$ 0.05, ****p $\le$ 0.0001. % cytotoxicity was calculated by normalizing the LDH release in the infected groups with uninfected cells (representing no cytotoxicity) and TritonX-100 treated cells (representing the highest cytotoxicity). (**B**) A549 cells were infected with the indicated bacteria for 6 h at MOI 50. Immunofluorescence was performed using anti-TOM20 antibody to stain mitochondria (red), anti-Flag antibody to stain OmpA (green) and DAPI to label nuclei (blue). Arrows indicate Flag positive OmpA_Ab_::Flag puncta (green) colocalizing with mitochondria (red). Scale bar represents 10 µm. (**C**) After 6 h of infection of A549 cells with the indicated bacteria at MOI 50, cellular fractionation was performed. Western blot analysis was done on the mitochondrial and cytosolic fractions using anti-Flag, anti-Tim23 and anti-ß-actin antibodies. **(D**) A549 cells were infected with *A. baumannii* (*Ab19606), E. coli* (*BW25113*) and *S. aureus* (*USA300*) for 6 h at MOI 50. Immunofluorescence was performed using anti-TOM20 antibody to stain mitochondria (red) and DAPI to stain the nucleus (blue). Scale bar represents 10 µm. (**E**) The scatter plots represent the quantification of mitochondrial area (red) and perimeter (orange). Error bars represent standard deviation, n = 47–113 cells. One-way ANOVA with Tukey’s multiple comparisons test ***p $\le$ 0.001, ****p $\le$ 0.0001. **(**F) A549 cells were infected with the indicated strains of *A. baumannii* for 6 h at MOI 50. Immunofluorescence was performed using anti-TOM20 antibody to stain mitochondria (red) and DAPI to stain the nucleus (blue). Scale bar represents 10 µm. (**G**) The scatter plots represent the quantification of mitochondrial area (red) and perimeter (orange). Error bars represent standard deviation, n = 39–55 cells. One-way ANOVA with Tukey’s multiple comparisons test *p $\le$ 0.05, **p $\le$ 0.01, ***p $\le$ 0.001, ****p $\le$ 0.0001. (**H**) A549 cells were infected with the indicated bacteria for 6 h at MOI 50. Immunofluorescence was performed using anti-TOM20 antibody to stain mitochondria (red) and DAPI to stain the nucleus (blue). Scale bar represents 10 µm. (**I**) The scatter plots represent the quantification of mitochondrial area (red) and perimeter (orange). Error bars represent standard deviation, n = 55–115 cells. One-way ANOVA with Tukey’s multiple comparisons test ****p $\le$ 0.0001. Mitochondrial area and perimeter quantifications were performed using an unbiased automated CellProfiler pipeline (see “Materials and methods” section for details). All the experiments shown here were performed three times independently.

Since we observed an important role of OmpA_Ab_ in mouse infection, we sought to understand the molecular mechanism of OmpA_Ab_ as a virulence factor. Epithelial cells (A549 and HeLa) and macrophages (RAW264.7) infected with wildtype *A. baumannii* exhibited enhanced cytotoxicity compared to *A. baumannii* ΔOmpA confirming earlier findings^20^ (Fig. 2A; Supplementary Fig. 2A–C). Two different *A. baumannii* strains (*Ab19606* and *Ab17978*) were tested to rule out any strain specific differences. Cells infected with the ΔOmpA + OmpA_Ab_::Flag complemented strain exhibited cytotoxicity comparable to wildtype *A. baumannii* infection demonstrating the importance of OmpA_Ab_ as a virulence factor (Fig. 2A).

Since *A. baumannii* is an extracellular bacterium and OmpA_Ab_ is required for inducing host cytotoxicity, we wondered if OmpA_Ab_ gains intracellular access during infection to induce cytotoxicity. To visualize the intracellular localization of OmpA_Ab_ during infection, we knocked-in a flag-tag on the endogenous OmpA_Ab_ in wildtype *A. baumannii* using genetic recombineering^26^. The OmpA_Ab_::Flag-tagged strain was verified by PCR and OmpA_Ab_::Flag expression was validated by western blotting (Supplementary Fig. 2D,E). Next, A549 cells were infected with the OmpA_Ab_::Flag-tagged strain and anti-flag antibody was used to perform immunofluorescence. Intriguingly, we detected Flag positive puncta directly colocalizing with mitochondria (Fig. 2B). We further validated these results biochemically by fractionating mitochondria after infecting A549 cells with the OmpA_Ab_::Flag-tagged strain. OmpA_Ab_::Flag was exclusively present in the mitochondrial fraction and not in the cytosolic fraction (Fig. 2C), confirming our imaging results. In addition, we observed a morphological disruption of the mitochondrial network in A549 cells infected with *A. baumannii.* The morphology of mitochondria in infected cells looked highly fragmented compared to uninfected cells (Fig. 2B,D,E). Mitochondrial fragmentation is not a general hallmark of bacterial infections as the infection of epithelial cells with other extracellular bacteria *E. coli* or *S. aureus* did not trigger mitochondrial fragmentation (Fig. 2D,E). To further confirm the specificity of this phenotype, A549 cells were infected with different strains of *A. baumannii* (lab adapted strains *Ab19606* and *Ab17978;* and the clinical isolate *Ab5075*). Interestingly all three *A. baumannii* strains induced mitochondrial fragmentation in A549 cells (Supplementary Fig. 2F,G). We then tested if mitochondrial fragmentation is specific to epithelial cells or if it also occurs in myeloid cells like macrophages upon *A. baumannii* infection. Murine RAW264.7 macrophages infected with *A. baumannii* revealed a similar pattern of mitochondrial fragmentation suggesting that *A. baumannii* infection induces a disruption in the mitochondrial network across different cell types (Supplementary Fig. 2H,I). A few bacteria including *Legionella pneumophila* and *Chlamydia trachomatis* are known to perturb the morphology of mitochondria as well as the endoplasmic reticulum (ER) and the Golgi apparatus thereby influencing the eukaryotic secretory pathway^25,28–31^. We performed immunofluorescence using the ER marker Calnexin and the Golgi marker GM130 and did not observe any differences in the ER and Golgi morphology upon infection with *A. baumannii* in A549 cells (Supplementary Fig. 2J,K)*.* Therefore, *A. baumannii* infection seems to alter only the mitochondrial network without affecting the morphology of the ER and the Golgi apparatus.

Further we wanted to assess the role of OmpA_Ab_ in the observed mitochondrial fragmentation phenotype. While the wildtype bacteria induced mitochondrial fragmentation, no difference was observed between the mitochondria of the uninfected cells and the cells infected with the isogenic ΔOmpA strains in the respective *Ab19606* and *Ab17978* genetic backgrounds (Fig. 2F,G; Supplementary Fig. 3A) confirming that OmpA_Ab_ is required for inducing mitochondrial fragmentation in host cells upon infection with *A. baumannii.* The mitochondrial fragmentation phenotype was fully restored in cells infected with the ΔOmpA + OmpA_Ab_::Flag complemented strain, further demonstrating that OmpA_Ab_ is required for inducing mitochondrial fragmentation (Fig. 2F,G). Additionally, we observed that 4 h post-infection was the earliest time-point where mitochondrial fragmentation was evident and it subsequently increased at 6- and 8-h post-infection with wildtype *A. baumannii* infection (Supplementary Fig. 3B). By contrast, the cells infected with *A. baumannii* ΔOmpA did not exhibit mitochondrial fragmentation suggesting that even after longer periods of infection of cells with *A. baumannii* ΔOmpA, mitochondrial fragmentation does not ensue which further substantiates the role of OmpA_Ab_ in inducing this phenotype (Supplementary Fig. 3C). Next, we tested if OmpA_Ab_ is sufficient to induce host cell mitochondrial fragmentation by expressing flag-tagged *OmpA*_*Ab*_ on a plasmid in *E. coli* (*E. coli OmpA*_*Ab*_). We validated the expression of flag-tagged *OmpA*_*Ab*_ in *E. coli* by western blotting and immunofluorescence (Supplementary Fig. 3D,E). Treatment of A549 cells with *E. coli OmpA*_*Ab*_ induced mitochondrial fragmentation while treatment with *E. coli* containing empty vector (*E. coli* EV) did not (Fig. 2H,I). The presence of endogenous *E. coli* OmpA (OmpA_Ec_) could occlude the expression of OmpA_Ab_ due to limited surface availability in the outer membrane of the *E. coli OmpA*_*Ab*_ strain. Therefore, we expressed *OmpA*_*Ab*_ on a plasmid in an *E. coli ΔOmpA*_*Ec*_ mutant (*E. coli ΔOmpA*_*Ec*_*,OmpA*_*Ab*_) and infected A549 cells with these bacteria. Similar mitochondrial fragmentation was detected in cells infected with *E. coli ΔOmpA*_*Ec*_*,OmpA*_*Ab*_ (Fig. 2I), indicating that OmpA_Ab_ is sufficient to induce mitochondrial fragmentation. Taken together, these data reveal that OmpA_Ab_ is necessary and sufficient to induce mitochondrial fragmentation.

**Figure 3:**
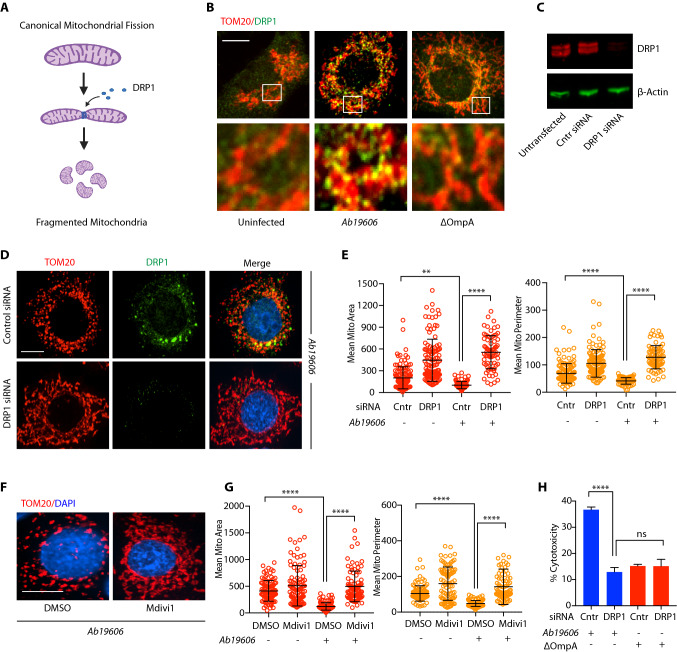
DRP1 is required for mitochondrial fragmentation upon *A. baumannii* infection. (**A**) The illustration represents the canonical mitochondrial fission pathway (created with BioRender.com). (**B**) A549 cells were infected with the indicated bacteria for 6 h at MOI 50. Immunofluorescence was performed using anti-TOM20 antibody to stain mitochondria (red) and anti-DRP1 antibody (green). Scale bar represents 10 µm. (**C**) Western blot on A549 cell lysates after indicated treatments. Anti-DRP1 antibody was used to examine DRP1 levels and ß-actin served as the loading control. (**D**) A549 cells treated with DRP1 siRNA or control (scrambled) siRNA were infected with wildtype *A. baumannii (Ab19606)* for 6 h at MOI 50. Immunofluorescence was performed using anti-TOM20 antibody to stain mitochondria (red), anti-DRP1 antibody (green) and DAPI to label the nucleus (blue). Scale bar represents 10 µm. (**E**) The scatter plots represent the quantification of mitochondrial area (red) and perimeter (orange). The experiment was performed in triplicates, n = 75–132 cells. Error bars represent standard deviation. One-way ANOVA with Tukey’s multiple comparisons test **p $\le$ 0.01, ****p $\le$ 0.0001 (**F**) A549 cells pre-treated with Mdivi1 (10 µM) or DMSO control were infected with wildtype *A. baumannii (Ab19606)* for 6 h at MOI 50. Immunofluorescence was performed using anti-TOM20 antibody to stain mitochondria (red) and DAPI to label the nucleus (blue). (**G**) The scatter plots represent the quantification of mitochondrial area (red) and perimeter (orange). The experiment was performed in triplicates, n = 88–138 cells. Error bars represent standard deviation. One-way ANOVA with Tukey’s multiple comparisons test ****p $\le$ 0.0001. (**H**) Cytotoxicity was assessed by LDH release assay after 6 h of infection in A549 cells treated with the indicated siRNA and bacteria. The experiments were done in triplicates. Error bars represent standard deviation. Two-tailed *p* value using unpaired t-test ****p $\le$ 0.0001. % cytotoxicity was calculated by normalizing the LDH release in the infected groups with uninfected/untreated cells (representing no cytotoxicity) and TritonX-100 treated cells (representing the highest cytotoxicity). Mitochondrial area and perimeter quantifications were performed using an unbiased automated CellProfiler pipeline (see “Materials and methods” section for details). All the experiments shown here were performed three times independently.

### DRP1 is required for mitochondrial fragmentation and cytotoxicity induced by *A. baumannii*

Mitochondrial fragmentation has been fairly well studied and the canonical pathway that mediates mitochondrial fragmentation is regulated by a GTPase domain-containing cytoplasmic protein belonging to the dynamin family of proteins called dynamin-related protein 1 or DRP1, also known as DNM1L. During mitochondrial fission that eventually leads to fragmentation of the mitochondrial network, DRP1 localizes to mitochondria and forms oligomeric structures wrapping around the mitochondrial fission sites and utilizes GTP to bring about a change in its conformation that ultimately causes scission of the mitochondrial tubules into fragmented mitochondria^22,32^ (Fig. 3A). Using immunofluorescence and confocal microscopy we observed enhanced DRP1 co-localization with mitochondria in A549 cells infected with wildtype *A. baumannii* compared to cells infected with *A. baumannii* ΔOmpA (Fig. 3B). To further validate its role, we knocked-down DRP1 by siRNA in A549 cells (Fig. 3C) and subsequently infected these cells with wildtype *A. baumannii.* Consistent with our immunofluorescence data, DRP1 loss completely abolished mitochondrial fragmentation upon *A. baumannii* infection (Fig. 3D,E). Concordantly, we also observed the absence of mitochondrial fragmentation when cells were treated with Mdivi-1 (a DRP1 inhibitor) prior to wildtype *A. baumannii* infection (Fig. 3F,G). Since we observed enhanced cytotoxicity with wildtype *A. baumannii* infection compared to *A. baumannii* ΔOmpA and cytotoxicity and cell death are associated with mitochondrial fragmentation, we asked if we can reverse cytotoxicity by preventing mitochondrial fragmentation and preserving mitochondrial integrity. We knocked-down DRP1 by siRNA in A549 cells and subsequently infected these cells with *A. baumannii* and monitored cytotoxicity. Consistent with our hypothesis we observed a significant reduction in cytotoxicity in cells treated with DRP1 siRNA relative to scrambled siRNA control upon infection with wildtype *A. baumannii* (Fig. 3H). There was no difference in cytotoxicity in DRP1 siRNA treated cells relative to scrambled siRNA control when infected with *A. baumannii* ΔOmpA suggesting that OmpA_Ab_ mediates its cytotoxic effects through mitochondrial fragmentation (Fig. 3H). Taken together these data suggest that OmpA_Ab_ induces canonical mitochondrial fragmentation which is driven by DRP1 and consistent with its effect on mitochondrial morphology, there is a corresponding increase in cytotoxicity in an OmpA_Ab_ dependent manner.

### ***A. baumannii*** infection perturbs mitochondrial physiology in an OmpA_Ab_-dependent manner

**Figure 4:**
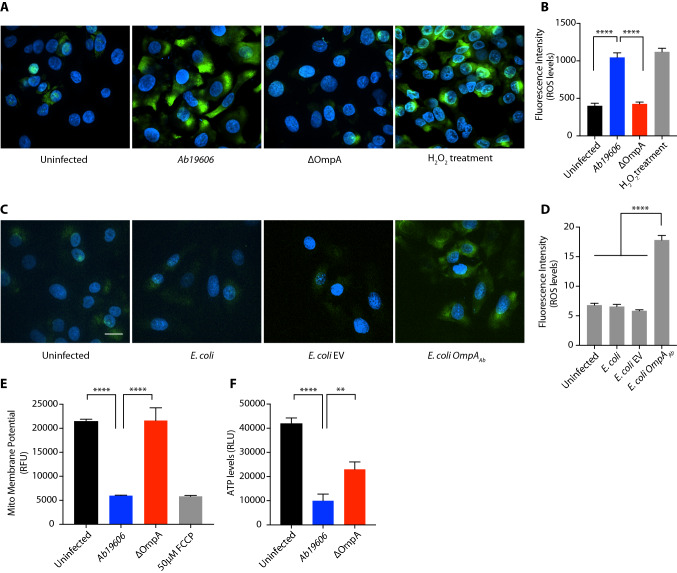
*A. baumannii* infection perturbs mitochondrial physiology in an OmpA_Ab_ dependent manner. (**A**) A549 cells were infected with the indicated bacteria for 6 h at MOI 50. The cells were treated with the CellROX reagent to examine ROS (green) and DAPI to label nuclei (blue). 30% (v/v) H_2_O_2_ treatment used at a final concentration of (1:1000) for 1 h at 37 °C served as positive control. Scale bar represents 20 µm. (**B**) The bar graph represents the quantification of ROS levels in A549 cells after the indicated infection/treatment. The experiment was done in triplicates, n = 90–103 cells. Error bars represent standard deviation. One-way ANOVA with Tukey’s multiple comparisons test ****p $\le$ 0.0001. (**C**) A549 cells were infected with the indicated bacteria for 6 h at MOI 50. The cells were treated with the CellROX reagent to examine ROS (green) and DAPI to label nuclei (blue). Scale bar represents 20 µm. (**D**) The bar graph represents the quantification of ROS levels in A549 cells after the indicated infection. The experiment was done in triplicates, n = 72–85 cells. Error bars represent standard deviation. One-way ANOVA with Tukey’s multiple comparisons test ****p $\le$ 0.0001. (**E**) TMRE assay to examine mitochondrial membrane potential in A549 cells after 24 h of infection with the indicated bacteria. The experiment was done in triplicates, Error bars represent standard deviation. One-way ANOVA with Tukey’s multiple comparisons test ****p $\le$ 0.0001. (**F**) ATP levels in A549 cells after 24 h of infection with the indicated bacteria. The experiment was done in triplicates. Error bars represent standard deviation. One-way ANOVA with Tukey’s multiple comparisons test **p $\le$ 0.01, ****p $\le$ 0.0001. All the experiments shown here were performed three times independently.

Having established that OmpA_Ab_ causes mitochondrial fragmentation, we next tested if OmpA_Ab_ also leads to perturbation of mitochondrial function. We examined reactive oxygen species (ROS) levels using CellROX fluorogenic probe and observed that green fluorescence indicative of ROS levels was significantly higher in wildtype *A. baumannii* infected A549 cells compared to cells infected with *A. baumannii* ΔOmpA (Fig. 4A,B). We next treated A549 cells with *E. coli OmpA*_*Ab*_ and *E. coli* EV. The green fluorescence was significantly stronger in cells infected with *E. coli OmpA*_*Ab*_ compared to cells infected with *E. coli* EV (Fig. 4C,D). These data show that OmpA_Ab_ is necessary and sufficient to increase the cellular ROS levels during infection. We then examined the effect of OmpA_Ab_ on mitochondrial membrane potential and ATP levels which are indicative of mitochondrial function. Tetramethylrhodamine ethyl ester (TMRE), a fluorescent cell-permeant dye that is sequestered in active functional mitochondria was used to assess mitochondrial membrane potential. We observed significantly reduced fluorescent signal, indicative of de-polarized mitochondria, in A549 cells infected with wildtype *A. baumannii* but not in cells infected with *A. baumannii* ΔOmpA (Fig. 4E)*.* Moreover, ATP levels in A549 cells infected with wildtype *A. baumannii* were also significantly reduced compared to cells treated with *A. baumannii* ΔOmpA (Fig. 4F). Together these results show that *A. baumannii* infection not only causes changes in mitochondrial morphology but also leads to functional defects in mitochondria as evidenced by enhanced levels of ROS, reduced mitochondrial membrane potential and reduced ATP levels. All of these effects on host mitochondria are dependent on OmpA_Ab_ suggesting that *A. baumannii* uses OmpA_Ab_ as a potent virulence factor that induces major perturbations in host cellular homeostasis.

### OMVs containing OmpA_Ab_ trigger mitochondrial fragmentation

**Figure 5:**
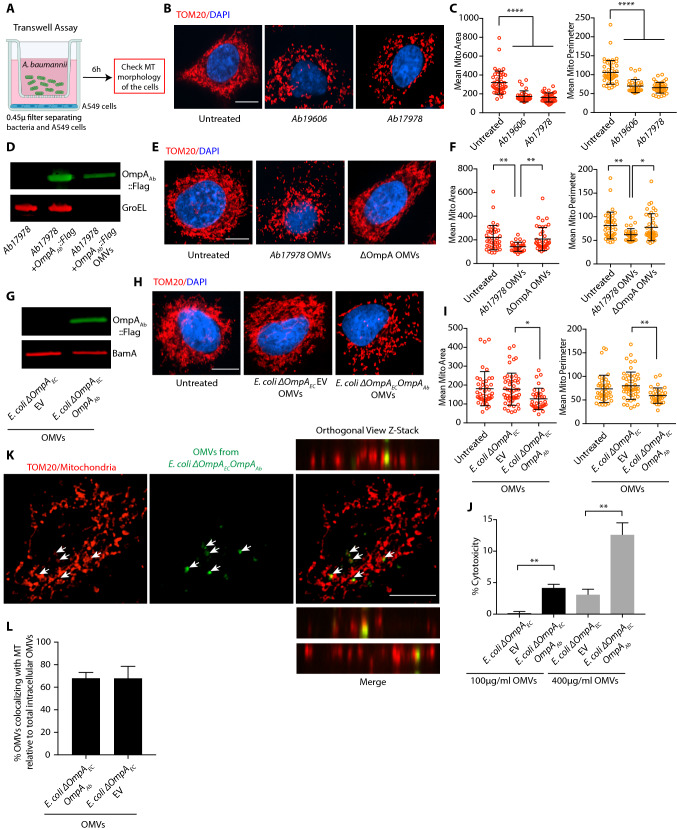
OMVs containing OmpA_Ab_ trigger mitochondrial fragmentation. (**A**) Illustration showing the transwell assay set-up (created with BioRender.com). (**B**) A549 cells seeded in the bottom chamber of the transwell plate were incubated with the indicated bacteria (corresponding to MOI 50) in the top chamber for 6 h. The bacteria were not in contact with the cells. Immunofluorescence was performed using anti-TOM20 antibody to stain mitochondria (red) and DAPI to stain the nucleus (blue). Scale bar represents 10 µm. (**C**) The scatter plots represent the quantification of mitochondrial area (red) and perimeter (orange). Error bars represent standard deviation, n = 32–46 cells. One-way ANOVA with Tukey’s multiple comparisons test ****p $\le$ 0.0001. (**D**) Western blot analysis of indicated bacterial lysates and OMVs using anti-Flag and anti-GroEL antibodies. (**E**) A549 cells were treated with 400 µg/ml of the indicated OMVs for 6 h. Immunofluorescence was performed using anti-TOM20 antibody to stain mitochondria (red) and DAPI to stain the nucleus (blue). Scale bar represents 10 µm. (**F**) The scatter plots represent the quantification of mitochondrial area (red) and perimeter (orange). Error bars represent standard deviation, n = 35–40 cells. One-way ANOVA with Tukey’s multiple comparisons test *p $\le$ 0.05, **p $\le$ 0.01. (**G**) Western blot analysis of indicated OMVs using anti-Flag and anti-BamA antibodies. (**H**) A549 cells were treated with 400 µg/ml of the indicated OMVs for 6 h. Immunofluorescence was performed using anti-TOM20 antibody to stain mitochondria (red) and DAPI to stain the nucleus (blue). Scale bar represents 10 µm. (**I**) The scatter plots represent the quantification of mitochondrial area (red) and perimeter (orange). Error bars represent standard deviation, n = 37–50 cells. One-way ANOVA with Tukey’s multiple comparisons test *p $\le$ 0.05, **p $\le$ 0.01. (**J**) Cytotoxicity was assessed by LDH release assay after 6 h of OMV treatment in A549 cells. The experiments were done in triplicates. Error bars represent standard deviation. Two-tailed *p* value using unpaired t-test **p $\le$ 0.01. % cytotoxicity was calculated by normalizing the LDH release in the treated groups with untreated cells (representing no cytotoxicity) and TritonX-100 treated cells (representing the highest cytotoxicity). (**K**) A549 cells were treated with 400 µg/ml of the indicated OMVs (fluorescently labelled with Vybrant Dio) for 6 h. Immunofluorescence was performed using anti-TOM20 antibody to stain mitochondria (red) and OMVs were labelled in green. Z-stack confocal imaging was performed on the cells, orthogonal views presented here. Arrows indicate OMVs (green) colocalizing with mitochondria (red). Scale bar represents 10 µm. (**L**) The bar graph represents the % of indicated OMVs colocalizing with mitochondria. The cells were stained with phalloidin and intracellular OMVs were scored for their colocalization with mitochondria or not. Error bars represent standard deviation. Mitochondrial area and perimeter quantifications were performed using an unbiased automated CellProfiler pipeline (see “Materials and methods” section for details). All the experiments shown here were performed three times independently.

To explore the mechanistic details of how OmpA_Ab_ triggers mitochondrial fragmentation, we first asked if bacterial contact with the host cells or bacterial internalization within the host cells are required to trigger mitochondrial fragmentation. To address this question, we used transwell plates where A549 cells were seeded in the bottom chamber while *A. baumannii* was added in the top chamber; these two compartments were separated by a 0.45 µm filter that prevented direct contact between the bacteria and the epithelial cells (Fig. 5A). The cells that were incubated with bacteria in the transwell plate exhibited mitochondrial fragmentation suggesting that bacterial internalization or bacterial contact with epithelial cells are not required to induce this phenotype (Fig. 5B,C). These data also indicate that OmpA_Ab_ is able to pass through the 0.45 µm filter to interact with host cells and brings about mitochondrial fragmentation. This led us to wonder if OMVs, which are vesicles shed by all Gram-negative bacteria, could be the potential carriers of OmpA_Ab_ since OMVs are predominantly loaded with outer membrane proteins^33,34^ and given their size (typically 20–200 nm)^34^, they would easily pass through the 0.45 µm transwell filter. Using a standard OMV isolation protocol (see “Materials and methods” section) we isolated OMVs from the OmpA_Ab_::Flag-tagged strain (described in Fig. 2B) and different control strains. The presence of OMVs from each preparation was confirmed by visualization using electron microscopy (Supplementary Fig. 4C). For the OmpA_Ab_::Flag-tagged strain, the presence of OmpA_Ab_ in the isolated OMVs was confirmed by western blotting (Fig. 5D). OmpA_Ab_ was exclusively membrane associated and not in the lumen of OMVs as assessed by the proteinase K protection assay (Supplementary Fig. 4A,B). Next, we treated A549 cells with the isolated OMVs (400 µg/ml; based on BCA protein estimation assay). Consistent with our previous results, treatment of A549 cells with OMVs isolated from wildtype *A. baumannii* induced mitochondrial fragmentation but not OMVs isolated from *A. baumannii* ΔOmpA (Fig. 5E,F).

To ascertain the sufficiency of OmpA_Ab_ in inducing mitochondrial fragmentation, OMVs from *E. coli ΔOmpA*_*Ec*_*,OmpA*_*Ab*_ and *E. coli ΔOmpA*_*Ec*_ EV were isolated and verified by visualization using electron microscopy (Supplementary Fig. 4C) and western blotting using an antibody against BamA, an outer membrane protein in *E. coli* (Fig. 5G). The presence of OmpA_Ab_ only in the OMV preparations from *E. coli ΔOmpA*_*Ec*_*,OmpA*_*Ab*_ but not from *E. coli ΔOmpA*_*Ec*_ EV was confirmed by western blotting for the FLAG tag that is fused to the OmpA_Ab_ protein; the FLAG signal was only present in the OMV preparation from *E. coli ΔOmpA*_*Ec*_*,OmpA*_*Ab*_ and not in the OMV preparation from *E. coli ΔOmpA*_*Ec*_ EV (Fig. 5G). Furthermore, contamination of OMVs with bacterial inner membrane was ruled out by western blotting using an antibody against MsbA, an inner membrane protein (Supplementary Fig. 4D). We then treated A549 cells with these OMVs (400 µg/ml; based on BCA protein estimation assay) and observed mitochondrial fragmentation in cells treated with OMVs from *E. coli ΔOmpA*_*Ec*_*,OmpA*_*Ab*_ (Fig. 5H,I). By contrast, there was no difference observed between the mitochondria of untreated cells and the cells treated with OMVs isolated from *E. coli ΔOmpA*_*Ec*_ EV (Fig. 5H,I). We also assessed cytotoxicity and found that the presence of OmpA_Ab_ in OMVs increased cytotoxicity in a concentration dependent manner (Fig. 5J). Together these data indicate that OmpA_Ab_ is necessary and sufficient to cause mitochondrial perturbation in the context of infection by intact bacterial cells (Fig. 2F–I) as well as by its presence in OMVs (Fig. 5E,F,H,I).

We next monitored fluorescently-labelled OMVs intracellularly in host epithelial cells. Isolated OMVs from *E. coli ΔOmpA*_*Ec*_*,OmpA*_*Ab*_ were labelled with the fluorescent dye Vybrant-DiO and then incubated with A549 cells (400 µg/ml; based on BCA protein estimation assay) for 6 h. Fluorescently-labelled OMVs could be detected within A549 cells after 6 h and intriguingly a large proportion of OMVs directly colocalized with mitochondria as revealed by confocal Z-stack microscopy (Fig. 5K; Supplementary Fig. 4E). We next asked if the presence of OmpA_Ab_ was required for the localization of OMVs to mitochondria. Interestingly, OMVs from *E. coli ΔOmpA*_*Ec*_ EV also colocalized with mitochondria suggesting that mitochondrial localization of OMVs did not depend on the presence of OmpA_Ab_ (Fig. 5L; Supplementary Fig. 4E). However, as described earlier mitochondrial fragmentation was totally dependent on the presence of OmpA_Ab_ in OMVs (Fig. 5H,I). Collectively, the data suggest that as an extracellular pathogen, *A. baumannii* utilizes OMVs as a delivery mechanism to target OmpA_Ab_ into host cells to induce mitochondrial fragmentation that is dependent on DRP1.

### OmpA_Ab_ induces mitochondrial fragmentation in vivo

**Figure 6:**
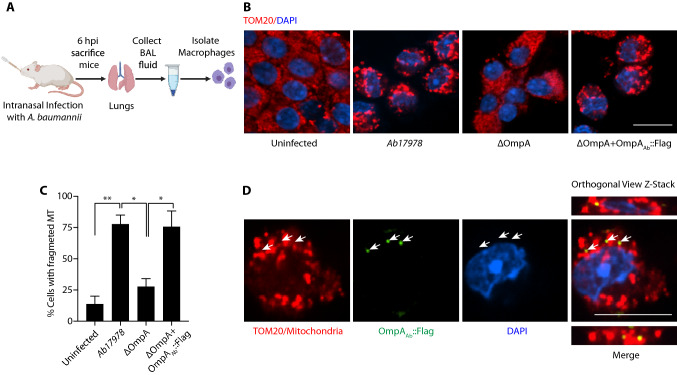
OmpA_Ab_ induces mitochondrial fragmentation in vivo*.* (**A**) Illustration of the mouse infection (created with BioRender.com). (**B**) Neutropenic BALB/c mice were infected intranasally with 10^7^ colony forming units (CFUs) of the indicated bacteria. Six hours post-infection bronchoalveolar lavage (BAL) fluid was collected from the trachea of mice. Alveolar macrophages were isolated and immunofluorescence was performed using anti-TOM20 antibody to stain mitochondria (red) and DAPI to stain nuclei. Scale bar represents 10 µm. (**C**) Quantification of cells with fragmented mitochondria. The cells displaying a discontinuous segmented mitochondrial network were scored as fragmented. The quantification was performed in a blinded manner. Error bars represent standard deviation, n = 157–521 cells. One-way ANOVA with Tukey’s multiple comparisons test *p $\le$ 0.05, **p $\le$ 0.01. (**D**) Immunofluorescence was performed on the alveolar macrophages isolated from the BAL fluid from mice infected with ΔOmpA + OmpA_Ab_::Flag complemented strain using anti-TOM20 antibody to stain mitochondria (red), anti-Flag antibody to stain OmpA::Flag (green) and DAPI to stain the nucleus (blue). Arrows indicate OmpA_Ab_::Flag (green) colocalization with mitochondria (red). Scale bar represents 10 µm.

Having established the role of OmpA_Ab_ in mediating mitochondrial fragmentation in vitro, we wondered about the relevance of our findings in an in vivo infection. We infected BALB/c mice intranasally with 10^7^ CFUs of wildtype *A. baumannii Ab17978*, isogenic *A. baumannii* ΔOmpA and the ΔOmpA + OmpA_Ab_::Flag complemented strain. After 6 h of infection, we sacrificed the animals and collected the bronchoalveolar lavage (BAL) fluid from the trachea and isolated alveolar macrophages for immunofluorescence to examine their mitochondrial morphology (Fig. 6A). We observed significant mitochondrial fragmentation in macrophages isolated from the BAL fluid of mice infected with wildtype *A. baumannii Ab17978* and the ΔOmpA + OmpA_Ab_::Flag complemented strain (Fig. 6B,C). Macrophages isolated from the BAL fluid of mice infected with *A. baumannii* ΔOmpA displayed mitochondrial morphology similar to uninfected mice suggesting that OmpA_Ab_ is required for inducing mitochondrial fragmentation in murine alveolar macrophages during a mouse lung infection (Fig. 6B,C). We further followed the localization of OmpA_Ab_::Flag in alveolar macrophages from the BAL fluid and observed OmpA_Ab_::Flag positive puncta colocalizing with mitochondria (Fig. 6D), similar to our initial observations in epithelial cells (Fig. 2B). Taken together these results suggest that similar to our in vitro data, OmpA_Ab_ targets mitochondria and induces mitochondrial fragmentation in murine macrophages upon *A. baumannii* infection.

## Discussion

A handful of primarily intracellular bacteria have been reported to perturb mitochondrial functions to disrupt host cell homeostasis^24^. In this study we describe how the extracellular bacterial pathogen *A. baumannii* uses the protein OmpA_Ab_ to induce significant changes in mitochondrial morphology and function ultimately leading to host cell death and pathogenesis. Our results provide a mechanistic framework and suggest in vivo relevance to the previously reported observation of the localization of recombinant OmpA_Ab_ protein to mitochondria^20^. We observed that *A. baumannii* infection induces mitochondrial fragmentation in host epithelial cells and macrophages in an OmpA_Ab_ dependent manner. Furthermore, OmpA_Ab_ also caused mitochondrial fragmentation in alveolar macrophages in a mouse lung infection model. Three genetically distinct isolates of *A. baumannii* including the clinically isolated strain *Ab5075* cause mitochondrial fragmentation in host cells, demonstrating that this is a clinically relevant and conserved virulence mechanism of *A. baumannii*. OmpA_Ab_ activates the canonical mitochondrial fragmentation pathway in lung epithelial cells which is driven by the host GTPase protein DRP1. Furthermore, OmpA_Ab_ induces higher levels of ROS, dissipation of mitochondrial membrane potential and a significant reduction in ATP levels all indicative of disrupted mitochondrial function.

One of the open questions in the field is how extracellular bacteria can potentially target mitochondria and furthermore, it is also unknown if this virulence strategy is relevant in vivo*.* We describe the mechanism of OmpA_Ab_ targeting mitochondria via OMVs released by *A. baumannii* and provide evidence that OmpA_Ab_ is required for mitochondrial disruption in vivo*.* OMVs are secreted by all Gram-negative bacteria and their major components include the outer membrane and periplasmic proteins. Additionally, extracellular bacteria can secrete OMVs carrying toxins and virulence factors that can penetrate the host cells and cause damage without the actual bacteria getting internalized into host cells^34–36^. In accord with this we observed that physically separating host epithelial cells from *A. baumannii* did not prevent mitochondrial fragmentation and that the treatment of host epithelial cells with OMVs containing OmpA_Ab_ was sufficient to induce mitochondrial fragmentation suggesting that OMVs carry OmpA_Ab_ into cells where it causes mitochondrial fragmentation. OMVs were seen to colocalize directly with mitochondria. Furthermore, OmpA_Ab_ positive puncta colocalized with mitochondria in alveolar macrophages derived from *A. baumannii* infected mice highlighting the relevance of our findings in *A. baumannii* pathogenesis in vivo. Since OmpA_Ab_ is in the membrane of OMVs, OMV-mitochondria colocalization potentially creates an interaction between OmpA_Ab_ and mitochondria that might be required for the induction of mitochondrial fragmentation. Interestingly, a recent study reported the targeting of *Neisseria gonorrhoeae* outer membrane porin protein PorB to mitochondria via OMVs suggesting that the strategy of targeting mitochondria and perturbing mitochondrial functions might be a conserved virulence mechanism across bacteria^37^. Even though it is believed that OMVs are targeted to lysosomes for degradation, several studies have shown the presence of OMVs in the cytoplasm of host cells^36–38^. In agreement with these studies, we also observed OMVs in the cytoplasm colocalizing with mitochondria. Yet, it is not completely understood how the presence of OmpA_Ab_ in OMVs induces mitochondrial fragmentation. It could be a direct insertion of the porin channel into mitochondria which leads to the release of cytochrome C into the cytoplasm thus disrupting mitochondrial function and leading to subsequent cell death. Alternatively, it could be an interaction of OmpA_Ab_ with mitochondrial protein/s that activates the DRP1 mediated mitochondrial fission pathway leading to fragmented mitochondrial network and cell death. Future studies to determine what host-cell proteins interact with OmpA_Ab_, and what structural features of OmpA_Ab_ are recognized and/or required for the phenotype will provide even greater mechanistic resolution of this process. We are intrigued by the fact that *E. coli* OmpA has none of the pathogenic functions of OmpA_Ab_, yet OmpA_Ab_ expressed in *E. coli* can bestow its heterologous host with the molecular phenotypes characteristic of OmpA_Ab_. It is tempting to speculate that the pathogenic function of OmpA_Ab_ described here may be a conserved mechanism of pathogenesis in other important human bacterial pathogens from the Moraxellaceae Family (e.g. *Moraxella catarrhalis*) and the *Pseudomonadales* Order (e.g. *Pseudomonas aeruginosa*).

*A. baumannii* infection often leads to severe pneumonia in immunocompromised people^39^. In the later stages of infection there is a disruption of the lung epithelial barrier due to cell death which allows the bacteria access to blood and causes the infection to spread to other organs, a critical condition called bacteremia, which eventually leads to sepsis^2,39,40^. We studied bacterial dissemination to different organs using wildtype *A. baumannii* and *A. baumannii* ΔOmpA in vivo*.* Using a murine lung infection model, we show that OmpA_Ab_ is required for bacterial colonization and dissemination to other organs demonstrating its crucial role in driving bacteremia during *A. baumannii* infections. Our work is in agreement with previous observations made in patients with *A. baumannii* infection where bacteremia and pneumonia were positively correlated with higher expression of OmpA_Ab_^14^. It has also been shown that the loss of OmpA_Ab_ makes *A. baumannii* susceptible to human serum which could also explain why *A. baumannii* ΔOmpA is incapable of inducing bacteremia in patients^18^. Taken together our work complements previous findings and additionally provides mechanistic insights into the role of OmpA_Ab_, and the OMVs that carry it, as a major virulence factor in *A. baumannii* pathogenesis.

## Materials and methods

### Bacterial strains and culture conditions

The *A. baumannii* strains used in this study included the lab adapted strains *Ab19606*, *Ab17978* and the clinical isolate *Ab5075. S. aureus* strain USA300 and *E. coli* Bw25113 were also used in this study. All the bacterial strains used in the study are listed in Supplementary Table 1. Bacterial strains were obtained from the American Type Culture Collection (ATCC). Bacterial cultures were grown by picking a single bacterial colony from tryptic soy agar (TSA) plates into tryptic soy broth (TSB) medium and the cultures were grown overnight at 37 °C.

### Cell culture

A549 lung epithelial cells, HeLa cells and RAW264.7 macrophages were used in this study. A549 cells were cultured in RPMI containing 10% (v/v) fetal bovine serum (FBS), 2 mM glutamine and 10 mM HEPES buffer. HeLa cells and RAW264.7 macrophages were cultured in Dulbecco’s Modified Eagle Medium (DMEM) containing 10% (v/v) fetal bovine serum (FBS), 2 mM glutamine and 10 mM HEPES buffer (pH 7.2). The cells were passaged two to three times a week and were used for experiments at passages between 7 and 25.

### In vitro infections

For infections, monolayers of cells were seeded a night before at the respective density for each experiment (see below for details). Overnight bacterial cultures were started in TSB medium. On the day of the experiment, bacteria were back-diluted in fresh TSB medium and grown upto an OD of 0.5. The cultures were centrifuged at 4500 rpm (3900 g) for 10 min at room temperature in a Beckman Coulter Allegra X-22R centrifuge and the bacterial pellet was resuspended in cell culture medium (Roswell Park Memorial Institute Medium (RPMI)/DMEM depending on the cells used). Bacteria were added onto cells at a multiplicity of infection (MOI) of 50. The cells treated with the bacteria were briefly centrifuged at 250 g for 5 min in an Eppendorf benchtop centrifuge for the bacteria to settle onto cells. The cells were incubated for respective times (see figure legends for details) at 37 °C. For infections with *A. baumannii* and *E. coli,* mammalian cells were treated with bacteria for 6 h before harvesting the cells for analysis*.* For infections with *S. aureus,* the cells were treated with bacteria for 1 h, after which the cells were washed 3 times to remove the extracellular bacteria. The cells were cultured for another 5 h (a total of 6 h post-infection) in cell culture medium containing 100 µg/ml Gentamicin to kill the left over extra-cellular bacteria.

### Cloning and genetic recombineering

*OmpA*_*Ab*_ was cloned using standard cloning techniques into *Acinetobacter-E. coli* shuttle vector pWH1266^41^. Briefly, C-terminally flag-tagged *OmpA*_*Ab*_ was synthesized by Integrated DNA Technologies (IDT) and using PCR, terminal overhangs complementary to the vector pWH1266 were added. The vector was digested with BamH1 and the PCR amplified fragmented and the digested vector were ligated and finally transformed into DH5α competent cells and plated on LB plates containing 50 µg/ml carbenicillin for selection. Positive colonies were verified by PCR and Sanger sequencing.

Genetic recombineering to generate *A. baumannii* ΔOmpA and OmpA_Ab_::Flag tagged strains was performed following the established recombineering method for *A. baumannii*^26,42^*.* Briefly, for OmpA_Ab_::Flag strains constructs were designed using *OmpA*_*Ab*_ coding sequence with upstream and downstream regions along with gentamicin resistance sequence for selection. For generating *A. baumannii* ΔOmpA, a kanamycin cassette containing upstream and downstream regions of *OmpA*_*Ab*_ coding sequence were used. The constructs were synthesized by IDT and PCR amplification of the product was performed further. 5 µg of the amplified PCR products were electroporated into competent *A. baumannii* expressing Rec_AB_ recombinase previously induced by 2 mM isopropyl ß-d-1-thiogalactopyranoside (IPTG). After electroporation the bacteria were outgrown in a total of 4 ml TSB media containing 2 mM IPTG for 4 h. Finally the bacteria were pelleted at 3000 rpm (956 g), most of the supernatant was removed and the final leftover volume containing the bacteria was plated on TSB plates containing 7.5 µg/ml gentamicin (for OmpA_Ab_::Flag strains) and 50 µg/ml kanamycin (for *A. baumannii* ΔOmpA strain). Notably, it was challenging to generate the complemented strain in the *A. baumannii* 19606 genetic background. Despite multiple efforts, we were unable to complement *A. baumannii* ΔOmpA strain in the *A. baumannii* 19606 genetic background. Therefore, we show phenotypic rescue in *A. baumannii* 17978 genetic background. Of note, we did not observe any strain specific differences between *A. baumannii* 19606 and *A. baumannii* 17978 which were tested throughout this study.

### Immunofluorescence, confocal microscopy and imaging data analysis

For immunofluorescence 10^5^ cells/chamber were seeded in a 4 chamber slide a night before the experiment. The next day after the experimental treatment, the cells were washed twice with PBS and fixed with 4% (v/v) paraformaldehyde (PFA) and then permeabilized with PBS containing 0.1% (v/v) triton-X 100 for 15 min at room temperature. After blocking in 1% (w/v) bovine serum albumin (BSA) for an hour at room temperature, the samples were treated with primary antibody (diluted in BSA at 1:200). After 1 h of incubation with the primary antibody at room temperature, the samples were washed three times with PBS and treated with fluorescently labelled (Alexa488 or Alexa594) secondary antibodies for one-hour room temperature. The samples were then mounted with ProLong Gold mounting medium containing DAPI. Antibodies used for immunofluorescence: Tom20 (Proteintech, 11802-1-AP), DRP1 (Novus, NB110-55288), Flag (M2, Sigma Aldrich).

For all the imaging Nikon Ti-E spinning disk confocal microscope was used. All the images were acquired using the 100X oil immersion objective. Image analysis and processing was performed using Fiji open source software. Mitochondrial imaging data analysis and quantification were performed in an unbiased automated way using an open source software called CellProfiler^43^. A CellProfiler pipeline was designed to capture mitochondria and the software added artificial masks and calculated area and perimeter of mitochondria from those masks. The quantification of mitochondrial fragmentation in alveolar macrophages from mouse BAL fluid was done as described before^44^. Briefly, the cells with a continuous peri-nuclear mitochondrial network were scored as normal while the cells displaying a discontinuous segmented peri-nuclear mitochondrial network were scored as fragmented. The quantification of mitochondrial fragmentation was performed in a completely blinded manner.

### Cytotoxicity analysis

Supernatants from 10^5^ cells treated with bacteria or OMVs were collected at specific time points (see figure legends). Levels of released LDH in the supernatants was assessed using the cytotoxicity kit **(**11644793001 Roche). Detergent induced cell lysis was used as 100% cytotoxicity and untreated cells were used as background. Relative cytotoxicity was calculated by comparing treated cells with detergent lysed cells and the background from untreated cells was subtracted.

### Western blotting

10^6^ cells/well were washed twice with PBS. The cells were lysed in 150 µl of RIPA buffer containing protease and phosphatase inhibitors (ThermoFisher Scientific). BCA protein estimation (ThermoFisher Scientific) was performed to ascertain the protein concentration. Standard western blot protocol was followed further^45^. Briefly, the lysates were mixed with SDS-PAGE sample buffer, boiled at 95 °C and 20 µg protein was loaded on an SDS-PAGE precast gel (Invitrogen). The gel was blotted onto a nitrocellulose membrane and blocked for an hour in the intercept blocking buffer (LI-COR) at room temperature. Primary antibodies were used at 1:1000 dilution and incubated overnight. The next day after three washes in tris-buffered saline with 0.1% (v/v) tween 20 detergent (TBST), secondary antibodies were added at 1:10,000 dilution followed by an hour of incubation at room temperature. The membranes were imaged with LI-COR Odyssey CLx imaging system. For western blots using bacterial lysates, log phase bacterial cultures were centrifuged down at 10,000 rpm (10,621 *g*) for 10 min in an Eppendorf benchtop centrifuge and resuspended in sample buffer. The samples were boiled at 95 °C and 20 µg protein was loaded on an SDS-PAGE precast gel (Invitrogen). Antibodies used for western blotting: DRP1 (Novus, NB110-55288), Flag (M2, Sigma Aldrich), β-Actin (CST), GroEL (Enzo, ADI-SPP-610), MsbA (produced in-house), Tim23 (Proteintech, 11123-1-AP). Uncropped western blot images are presented in Supplementary Fig. 5.

### siRNA knockdown

10^5^ A549 cells/well were treated with 5 µM DRP1 siRNA (ON TARGETplus siRNA, Horizon Discovery). 5 µl of 5 µM siRNA (DRP1 or scrambled) was mixed with 95 µl serum free RPMI and further mixed with 100 µl of transfection reagent (5% v/v transfection formulation in serum free RPMI) and added onto cells. The cells were incubated for 48 h at 37 °C. Transfection efficiency was verified by western blotting.

### OMV isolation and fluorescent labeling

OMVs were isolated as described previously with a few modifications^46^. One liter overnight grown bacterial cultures were centrifuged at 12,000 rpm (24,470 *g*) for 30 min at 4 °C in a Thermo Scientific Sorvall Lynx 6000 centrifuge. The supernatant was filtered through a 0.45 µm PVDF filter. The filtered supernatant was centrifuged at 40,000 rpm (185,511 *g*) for 2 h at 4 °C in a Beckman Coulter Optima L-100 K ultracentrifuge. The supernatant was removed and the pellet containing OMVs was resuspended in PBS containing 200 mM NaCl, 1 mM CaCl2, and 0.5 mM MgCl2. Protein concentration in OMVs was assessed by the BCA assay (ThermoFisher Scientific). For fluorescent labeling, OMVs were treated with the fluorescent dye Vybrant DiO (ThermoFisher Scientific) at 1:100 and incubated at 37 °C for 20 min. After the labeling, the samples were centrifuged at 10,000 rpm (10621 g) in an Eppendorf benchtop centrifuge to remove excess dye using Micron 10 kDa centrifugal filter units following manufacturer's protocol. The samples were washed three times with PBS and centrifuged at 3000 rpm (956 *g*) in an Eppendorf benchtop centrifuge to remove the unattached dye.

### Mitochondrial assays: assessing ROS levels, mitochondrial membrane potential and ATP levels

ROS levels were assessed using the CellROX green reagent (ThermoFisher Scientific) following the manufacturer’s protocol. 30% v/v H_2_O_2_ used at a final concentration of 1:1000 for an hour at 37 °C served as the positive control. The cells were fixed with 4% v/v PFA, mounted with ProLong Gold mounting medium containing DAPI and imaged.

Mitochondrial membrane potential was examined using the TMRE based assay (ThermoFisher Scientific) following the manufacturer’s protocol. The assay was performed in a 96 well dish and the fluorescence was read using a BioTek microplate reader.

ATP levels were examined by using the ATPlite kit (Perkin-Elmer) following the manufacturer’s protocol. The assay was performed in white walled 96-well dishes and the luminescence was read using a BioTek microplate reader.

### Mitochondrial isolation

Mitochondrial isolation was carried out using the mitochondrial isolation kit for cultured cells (ThermoFisher Scientific). Briefly, 10^7^ cells after infection were washed three times with PBS and detached using TrypLE (ThermoFisher Scientific, Cat. No. 89874). The kit protocol was followed for cell fractionation. Mitochondrial and cytoplasmic fractions were collected at the end of the experiment and ran on an SDS-PAGE followed by western blot using antibodies Tim23 (Proteintech, 11123-1-AP) for the mitochondrial fraction and β-Actin (CST) for the cytoplasmic fraction.

### Mouse infection

#### Animal usage declaration

All animal procedures were conducted under protocols approved by the Genentech Institutional Animal Care and Use Committee in an Association for Assessment and Accreditation of Laboratory Animal Care (AAALAC)-accredited facility in accordance with the Guide for the Care and Use of Laboratory Animals and applicable laws and regulations.

#### Neutropenic lung infection model

Neutropenic lung infection model for *A. baumannii* was used for mouse infection studies^47^. 6–8 weeks old Female BALB/c mice (Charles River/Hollister) were rendered neutropenic by 2 intraperitoneal injections of Cytoxan (Baxter Health Care Corporation) at 150 mg/kg on Day-4 and 100 mg/kg on Day-1. On day 0, mice were infected intranasally with 50 ul log-phase grown *A. baumannii* at 10^7^ CFU/mouse. At 2- and 24-h post infection, bacterial load (CFUs) was determined in the lung, spleen, liver and kidneys through serial dilutions.

### Isolation of macrophages from bronchoalveolar lavage (BAL) fluid

Mouse infections were performed as described earlier. A total of 12 mice, three per group (groups: uninfected control, wildtype *A. baumannii Ab17978, A. baumannii* ΔOmpA and ΔOmpA + OmpA_Ab_::Flag) were used for the experiment. BAL fluid was collected from the trachea of mice 6-h post infection and was centrifuged at 3000 rpm (956 g) at 4 °C for 10 min to pellet the macrophages in an Eppendorf benchtop centrifuge. The macrophages were washed twice with PBS and fixed with 4% v/v PFA in PBS for 15 min at room temperature. After a couple of more washes, cytospin was used to deposit the macrophages evenly on a microscopic slide. The deposited macrophages were encircled with a PAP pen in order to retain liquid on them during the immunofluorescence steps. Standard immunofluorescence (as described above) was performed on the slide directly taking care not to dislodge the cells. The antibodies used for immunofluorescence were anti-TOM20 and anti-Flag antibodies. The samples were finally mounted with ProLong Gold mounting medium containing DAPI.

### Electron microscopy

The suspension of OMVs was adsorbed for 15 min to the surface of formvar and carbon coated transmission electron microscope (TEM) grids. After a shot rinse with distilled water, the sample was stained with 2% (v/v) uranyl acetate for 60 s and then air dried. Imaging was performed with a JEOL JEM-1400 TEM and a GATAN Ultrascan 1000 CCD camera at magnifications from 5000 × to 50,000 ×. Scale bars are indicated in the images.

## Supplementary Information

Supplementary figure legends.

Supplementary Information.
