# Supplementary material for: Outer membrane vesicles containing OmpA induce mitochondrial fragmentation to promote pathogenesis of Acinetobacter baumannii
Source: Sci Rep. 2021 Jan 12;11:618. doi: 10.1038/s41598-020-79966-9 (PMC7804284; doi:10.1038/s41598-020-79966-9)
Supplement: Supplementary file 1 — Supplementary figure legends. [file 41598_2020_79966_MOESM1_ESM.docx]

**Supplementary Figure Legends**

**Supplementary Figure 1.** (A) Schematic representation of the genetic recombineering strategy followed in *A. baumannii* (created with BioRender.com)*.* (B) Validation of *A. baumannii* ΔOmpA and ΔOmpA+OmpA_Ab_::Flag strains. Coomassie blue stained gel showing the absence of the OmpA_Ab_ band in *A. baumannii* ΔOmpA (middle lane) and the presence of the OmpA_Ab_ band in the complemented strain ΔOmpA+OmpA_Ab_::Flag. Note that OmpA_Ab_ band in the complemented strain ΔOmpA+OmpA_Ab_::Flag runs slightly higher than the wildtype OmpA_Ab_ band due to the presence of the Flag tag. (C) Validation of the protein expression of OmpA_Ab_::Flag by western blot using anti-Flag antibody and anti-GroEL antibody serving as loading control.

**Supplementary Figure 2.** (A) Cytotoxicity was assessed by LDH release assay in A549 cells infected with the indicated bacteria for the indicated times. The experiments were done in triplicates. Error bars represent standard deviation. Two-tailed p-value using unpaired t-test *p $\leq$ 0.05, ****p $\leq$ 0.0001. (B,C) Cytotoxicity was assessed by LDH release assay after 24 hours of infection with the indicated bacteria in HeLa cells (B) and RAW264.7 macrophages (C). The experiments were done in triplicates. Error bars represent standard deviation. Two-tailed p-value using unpaired t-test **p $\leq$ 0.01, ***p $\leq$ 0.001. (D) DNA gel showing ~ 500 bp shift in the OmpA_Ab_::Flag strain compared to the wildtype parent strain *Ab17978.* (E) Validation of the protein expression of OmpA_Ab_::Flag by western blot using anti-Flag antibody and anti-GroEL antibody serving as loading control. (F) A549 cells were infected with different strains of *A. baumannii* (lab adapted strains *Ab19606, Ab17978* and clinical isolate *Ab5075)* for 6 hours at MOI 50. Immunofluorescence was performed using anti-TOM20 antibody to stain mitochondria (red) and DAPI to stain the nucleus (blue). Scale bar represents 10 µm. (G) The scatter plots represent the quantification of mitochondrial area (red) and perimeter (orange). Error bars represent standard deviation, n = 32 - 69 cells. One-way ANOVA with Tukey’s multiple comparisons test *p $\leq$ 0.05, **p $\leq$ 0.01, ****p $\leq$ 0.0001. (H) RAW264.7 macrophages were infected with different strains of *A. baumannii* (lab adapted strains *Ab19606, Ab17978* and clinical isolate *Ab5075)* for 6 hours at MOI 50. Immunofluorescence was performed using anti-TOM20 antibody to stain mitochondria (red) and DAPI to stain the nucleus (blue). Scale bar represents 10 µm. (I) The scatter plots represent the quantification of mitochondrial area (red) and perimeter (orange). Error bars represent standard deviation, n = 35 - 70 cells. One-way ANOVA with Tukey’s multiple comparisons test *p $\leq0.05,$**p $\leq$ 0.01, ***p $\leq$ 0.001. (J,K) A549 cells were infected with the indicated strains of *A. baumannii* for 6 hours at MOI 50. Immunofluorescence was performed using anti-calnexin antibody to stain the ER (J), anti-GM130 antibody to stain the Golgi complex (K) and DAPI to label the nucleus (blue). Scale bars represent 20 µm. Mitochondrial area and perimeter quantifications were performed using an unbiased automated CellProfiler pipeline (see methods for details).

**Supplementary Figure 3. (**A) A549 cells were infected with the indicated strains of *A. baumannii* for 6 hours at MOI 50. Immunofluorescence was performed using anti-TOM20 antibody to stain mitochondria (red) and DAPI to stain the nucleus (blue). Scale bar represents 10 µm. (B,C) The scatter plots represent the quantification of mitochondrial area (red) and perimeter (orange). Error bars represent standard deviation, n = 52 - 79 cells. One-way ANOVA with Tukey’s multiple comparisons test **p $\leq$ 0.01, ***p $\leq$ 0.001. (D) Validation of the protein expression of OmpA_Ab_::Flag in the indicated bacteria by western blot using anti-Flag antibody and anti-GroEL antibody serving as loading control. (E) Immunofluorescence showing the expression of OmpA_Ab_::Flag in *E. coli*. Anti-Flag antibody was used to stain OmpA_Ab_::Flag (green) and DAPI was used to stain DNA (blue). Scale bar represents 2 µm. Mitochondrial area and perimeter quantifications were performed using an unbiased automated CellProfiler pipeline (see methods for details).

**Supplementary Figure 4.** (A,B) Proteinase K protection assay performed on OMVs from the indicated bacteria (schematic representation in panel A created with BioRender.com). (C) Negative stain electron microscopy was done on outer membrane vesicles (OMVs) isolated from the indicated bacteria. The size range of OMVs varied from 30 nm to 200 nm. Scale bar represents 100 nm. (D) Western blot analysis of the indicated bacterial lysates and OMVs using anti-MsbA and anti-BamA antibodies. (E) Immunofluorescence of A549 cells treated with fluorescently labelled OMVs from *E. coli ΔOmpA_Ec_* EV (green) and mitochondria stained with anti-TOM20 antibody (red). The OMV treatment was carried out for 6 hours. Arrows indicate OMVs (green) colocalizing with mitochondria (red). Scale bar represents 10 µm.

**Supplementary Figure 5.** (A-I) Uncropped western blot images used in this study.
