# Supplementary material for: Outer membrane vesicles containing OmpA induce mitochondrial fragmentation to promote pathogenesis of Acinetobacter baumannii
Source: Sci Rep. 2021 Jan 12;11:618. doi: 10.1038/s41598-020-79966-9 (PMC7804284; doi:10.1038/s41598-020-79966-9)
Supplement: Supplementary file 2 — Supplementary Information. [file 41598_2020_79966_MOESM2_ESM.pdf]

Outer membrane vesicles containing OmpA induce mitochondrial fragmentation to promote pathogenesis of *Acinetobacter baumannii*

Varnesh Tiku[1], Eric M. Kofoed[1], Donghong Yan[2], Jing Kang[2], Min Xu[2], Mike Reichelt[3], Ivan Dikic[1,4,5,6]* and Man-Wah Tan[1]*

[1] Department of Infectious Diseases, Genentech Inc., 1 DNA Way, South San Francisco, CA 94080, USA

[2] Department of Translational Immunology, Genentech Inc., 1 DNA Way, South San Francisco, CA 94080, USA

[3] Department of Pathology, Genentech Inc., 1 DNA Way, South San Francisco, CA 94080, USA

[4] Institute of Biochemistry II, Faculty of Medicine, Goethe University Frankfurt, Theodor-Stern- Kai 7, 60590 Frankfurt am Main, Germany

[5] Buchmann Institute for Molecular Life Sciences, Goethe University Frankfurt, Max-von-Laue-Str. 15, 60438 Frankfurt am Main, Germany

[6] Max Planck Institute of Biophysics, Max-von-Laue-Str. 3, 60438 Frankfurt am Main, Germany

* Corresponding authors (tan.man-wah@gene.com and dikic@biochem2.uni-frankfurt.de)

Supplementary Material

(Supplementary Figures 1 – 5; Supplementary Table 1)

Supplementary Figure 1

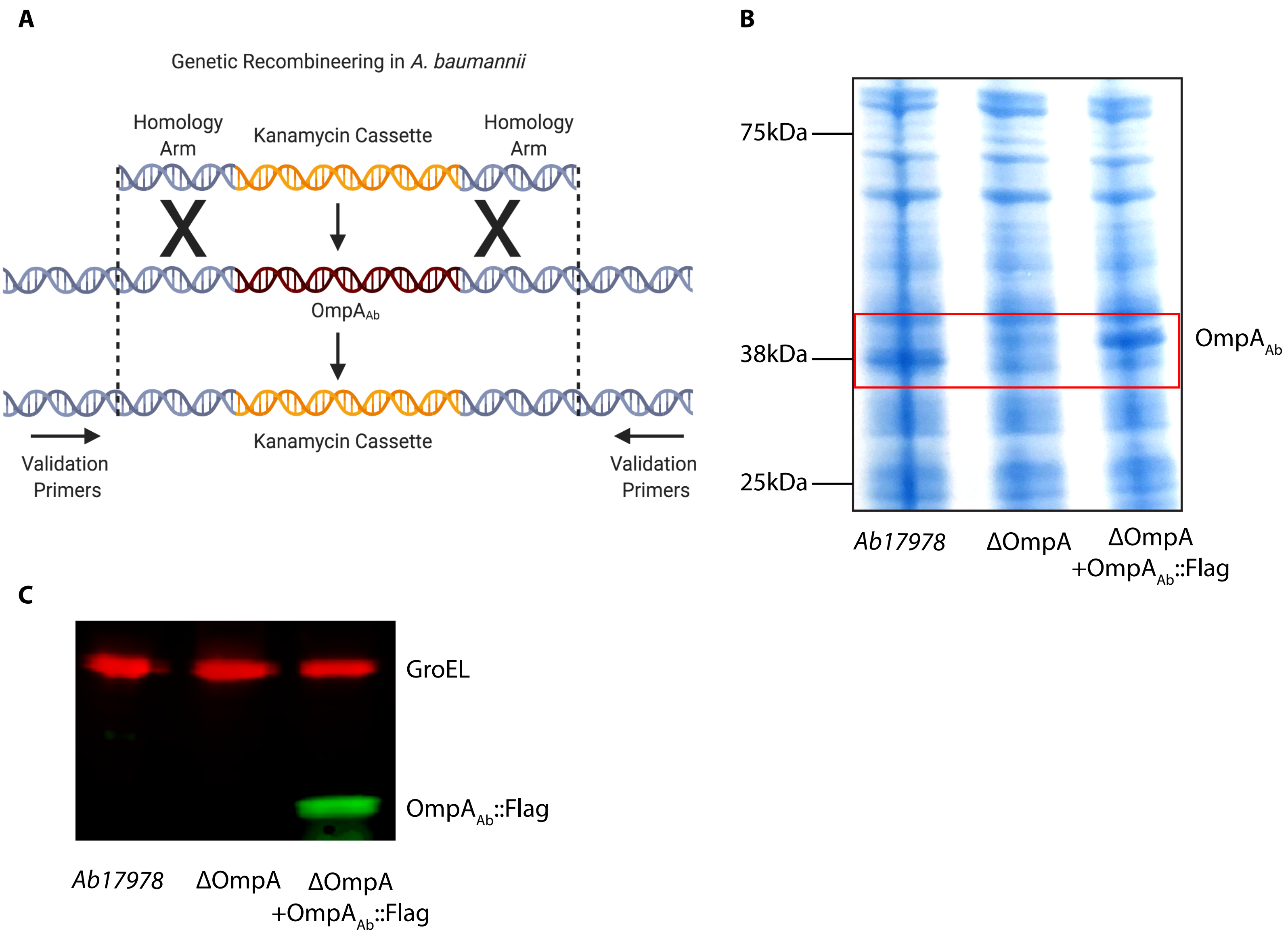

Supplementary Figure 2

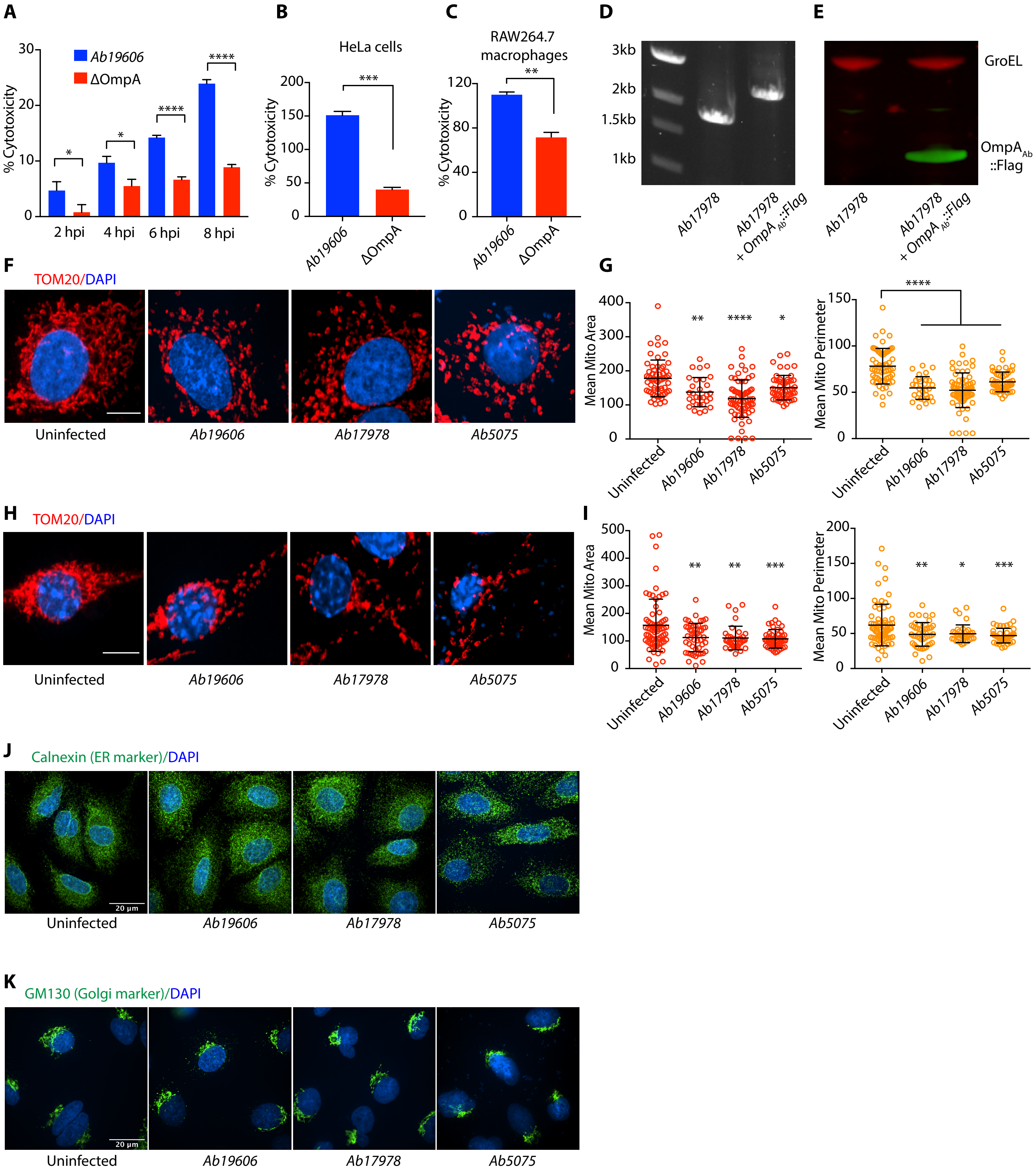

Supplementary Figure 3

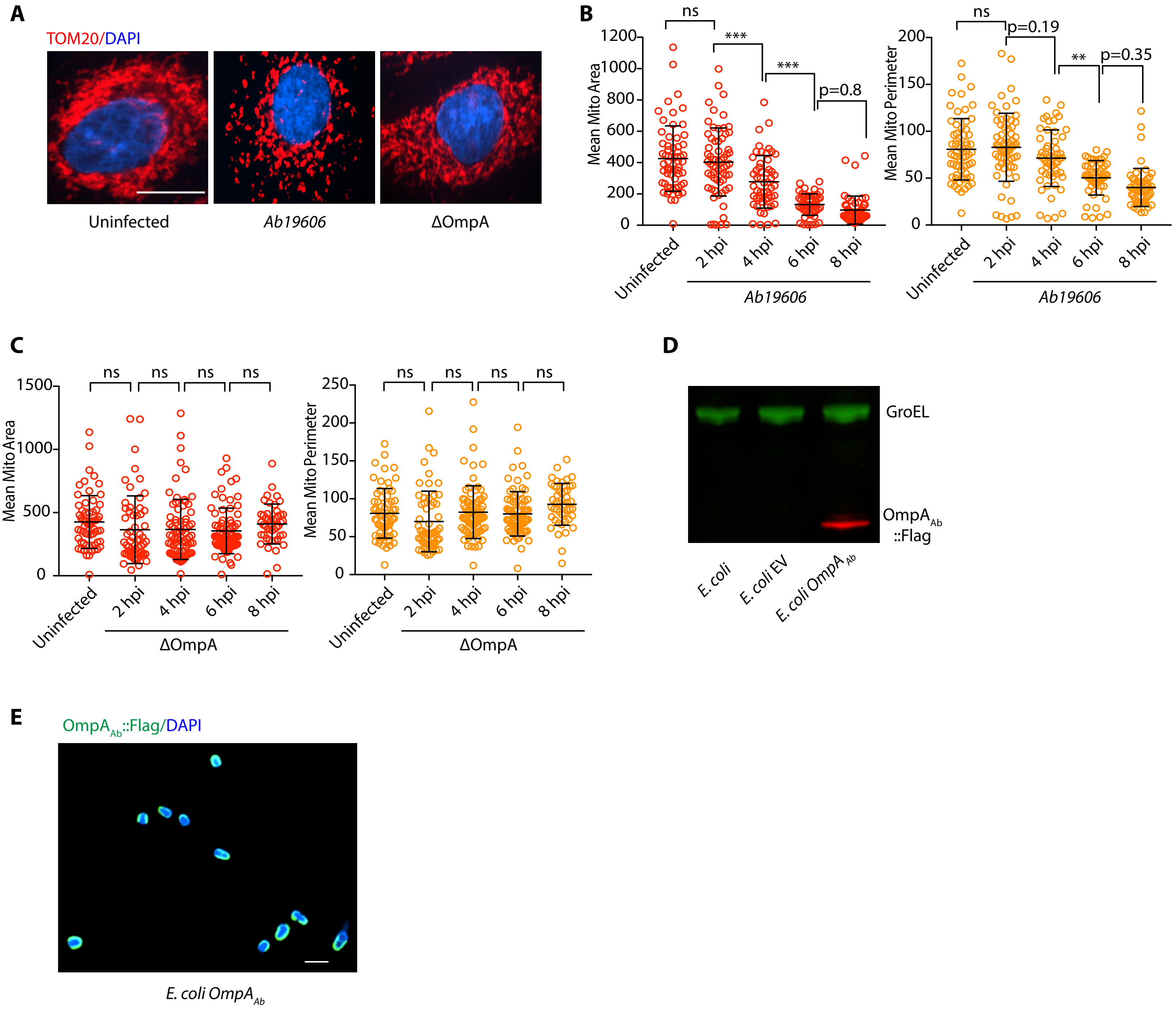

Supplementary Figure 4

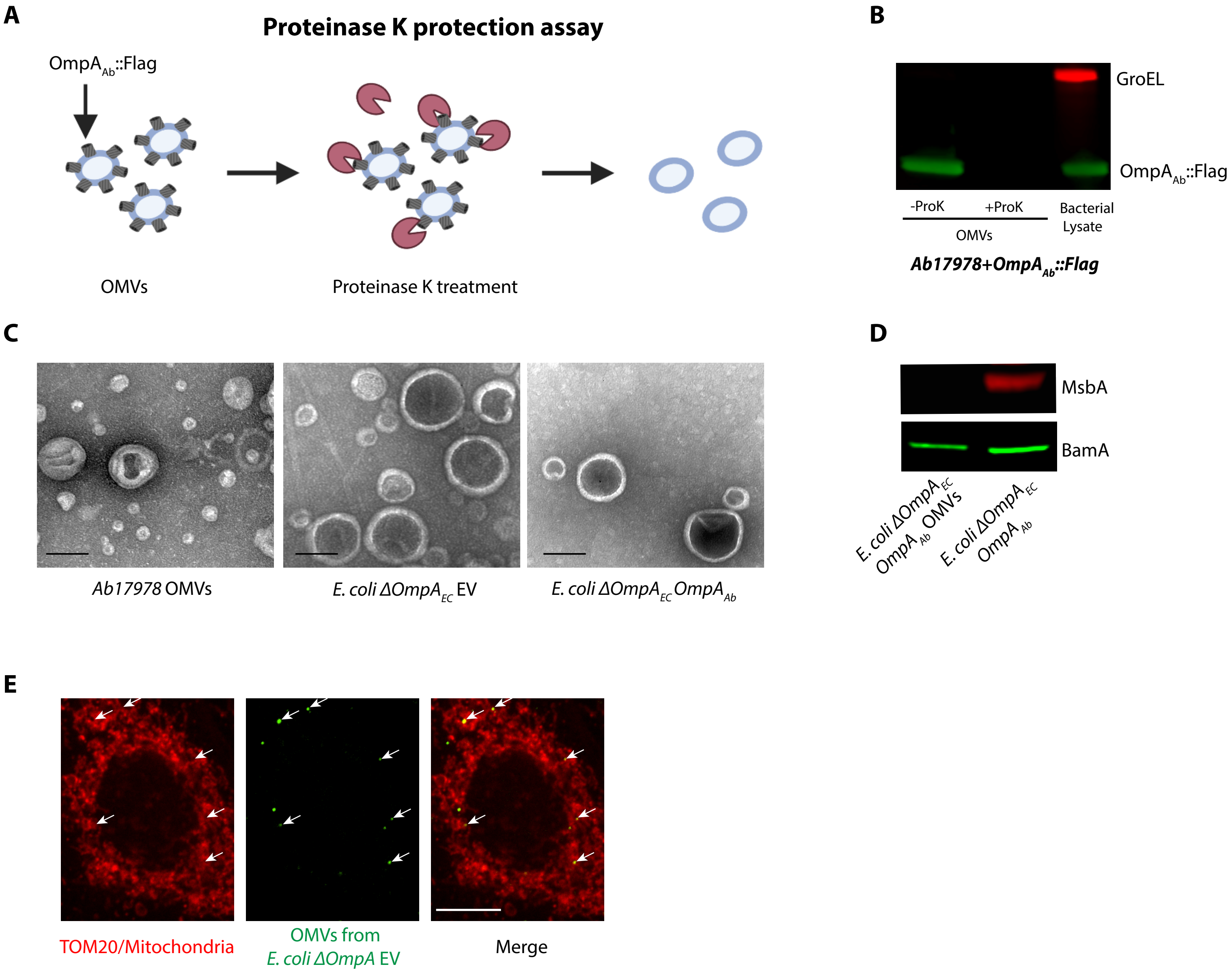

Supplementary Figure 5

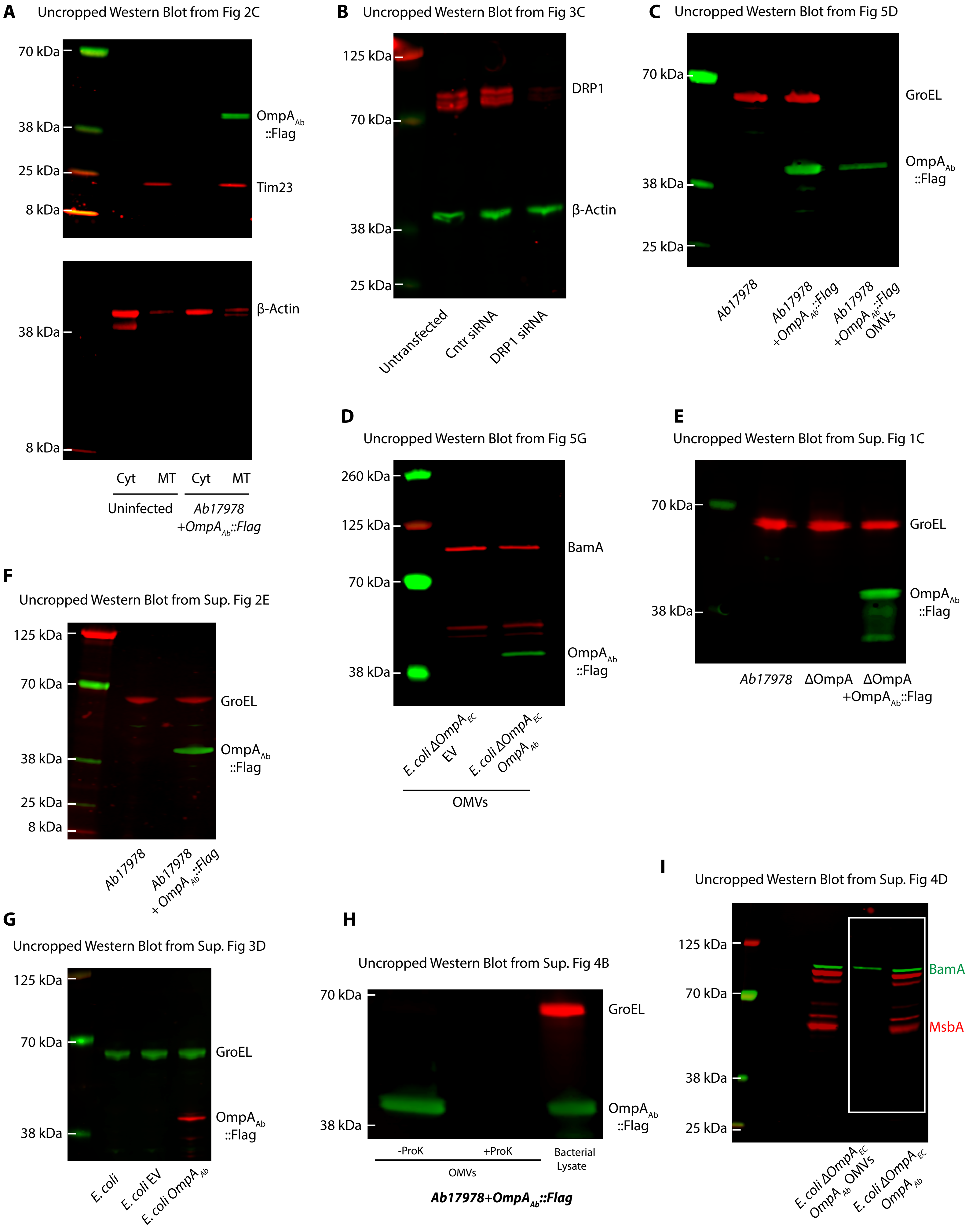

Supplementary Table 1: Bacterial strains used in the study

Bacterial species/strains	Genotype/Other information	Study Designation
A. baumannii Ab17978	Wildtype Ab17978	Wildtype or WT Ab17978
A. baumannii Ab17978 ΔOmpA	ΔOmpA	ΔOmpA
A. baumannii Ab17978 ΔOmpA complemented strain	ΔOmpA+$OmpA_{Ab}$::Flag (Integrated in the genome)	ΔOmpA+$OmpA_{Ab}$::Flag
A. baumannii Ab19606	Wildtype Ab19606	Wildtype or WT Ab19606
A. baumannii Ab19606 ΔOmpA	ΔOmpA	ΔOmpA
A. baumannii Ab5075 clinical isolate	Wildtype Ab5075	Ab5075
A. baumannii Ab17978	Wildtype Ab17978+$OmpA_{Ab}$::Flag (Integrated in the genome)	Ab17978+$OmpA_{Ab}$::Flag
E. coli BW25113	Wildtype *E. coli*	*E. coli*
E. coli BW25113	*E. coli* BW25113 + empty vector pWH1266	*E. coli* EV
E. coli BW25113 ΔOmpA	*E. coli* BW25113 ΔOmpA + empty vector pWH1266	*E. coli* $ΔOmpA_{EC}$ EV
E. coli BW25113	*E. coli* BW25113 + $OmpA_{Ab}$ in pWH1266	*E. coli* $OmpA_{Ab}$
E. coli BW25113 ΔOmpA	*E. coli* BW25113 ΔOmpA + $OmpA_{Ab}$ in pWH1266	*E. coli* $ΔOmpA_{EC}$, $OmpA_{Ab}$
S. aureus USA300	Wildtype *S. aureus* USA300	*S. aureus*
